# Local dose rate effects in implantable cardioverter–defibrillators with flattening filter free and flattened photon radiation

**DOI:** 10.1007/s00066-022-01911-8

**Published:** 2022-03-10

**Authors:** Benjamin Gauter-Fleckenstein, Erol Tülümen, Boris Rudic, Martin Borggrefe, Martin Polednik, Jens Fleckenstein

**Affiliations:** 1 Department of Radiation Oncology, University Medical Center Mannheim, University of Heidelberg, Theodor-Kutzer-Ufer 1–3, 68167 Mannheim, Germany; 2 I. Medizinische Klinik, University Medical Center Mannheim, University of Heidelberg, Mannheim, Germany; 3 Partner Site Heidelberg/Mannheim, German Center for Cardiovascular Research (DZHK), Mannheim, Germany

**Keywords:** Planning target volume, Sterotactic body radiotherapy, Cardiac implantable electronic devices, Flattening filter free radiation, Malfunction

## Abstract

**Purpose:**

In the beam penumbra of stereotactic body radiotherapy volumes, dose rate effects in implantable cardioverter–defibrillators (ICDs) may be the predominant cause for failures in the absence of neutron-generating photon energies. We investigate such dose rate effects in ICDs and provide evidence for safe use of lung tumor stereotactic radioablation with flattening filter free (FFF) and flattened 6 Megavolt (MV) beams in ICD-bearing patients.

**Methods:**

Sixty-two ICDs were subjected to scatter radiation in 1.0, 2.5, and 7.0 cm distance to 100 Gy within a 5 × 5 cm^2^ radiation field. Radiation was applied with 6 MV FFF beams (constant dose rate of 1400 cGy/min) and flattened (FLAT) 6 MV beams (430 cGy/min). Local dose rates (LDR) at the position of all ICDs were measured. All ICDs were monitored continuously.

**Results:**

With 6 MV FFF beams, ICD errors occurred at distances of 1.0 cm (LDR 46.8 cGy/min; maximum ICD dose 3.4 Gy) and 2.5 cm (LDR 15.6 cGy/min; 1.1 Gy). With 6 MV FLAT beams, ICD errors occurred only at 1 cm distance (LDR 16.8 cGy/min; 3.9 Gy). No errors occurred at an LDR below 7 cGy/min, translating to a safe distance of 2.5 cm (1.5 Gy) in flattened and 7 cm (0.4 Gy) in 6 MV FFF beams.

**Conclusion:**

A LDR in ICDs larger than 7 cGy/min may cause ICD malfunction. At identical LDR, differences between 6 MV FFF and 6 MV FLAT beams do not yield different rates of malfunction. The dominant reason for ICD failures could be the LDR and not the total dose to the ICD. For most stereotactic treatments, it is recommended to generate a planning risk volume around the ICD in which LDR larger than 7 cGy/min are avoided.

## Introduction

Considering stereotactic body radiotherapy (SBRT) and its effects on implantable cardioverter–defibrillators (ICDs), evidence is limited to case reports and mechanistic studies which describe such phenomena but fail to identify causes aside from neutron-generating photon energies.

Recently, we presented an investigation of radiation-induced effects on cardiac implantable electronic devices (CIEDs), which showed that volumetric modulated arc therapy (VMAT) without a flattening filter (flattening filter free; FFF) at 6 Megavolt (MV) with typical dose rates at the isocenter may be safely applied even at a distance (2.5 cm) close to the devices (specifically ICDs) [1]. Here, the emphasis was on clinical scenarios like normo- or slightly hypofractionated treatments of mediastinal and pelvic tumors (esophageal or central lung cancer and prostate carcinoma) and discrimination of CIED effects between neutron-generating and non-neutron-generating photon beams (6 MV vs. 10 MV and 18 MV). Dose rate effects (in non-neutron-generating beams) were not addressed.

Especially since the clinical introduction of FFF radiotherapy, high dose rates at the isocenter of up to 1500 cGy/min for 6 MV FFF and 2500 cGy/min for 10 MV FFF photon beams can be achieved. Although FFF is known [2] to generate less scattered radiation especially for low-modulated stereotactic body radiotherapy (SBRT; e.g., SBRT in the lung), it may result in undesirably high local dose rates (LDR) within the CIED in realistic clinical scenarios. AAPM TG-203 report [3], the most recent comprehensive review on this topic, states in this context that currently no evidence exists on safe application of FFF radiotherapy for patients with CIEDs and suggests an increased monitoring of CIEDs pre- and posttreatment, which increases the workload for healthcare providers. A recent review on 13 lung cancer SBRT cases and a phantom study with explanted ICDs showed that even though no CIED failures were reported on the historical SBRT cohort, in vitro data suggest inappropriate sensing (IS) starting at a dose rate (DR) of 1200 cGy/min (200 mGy/s) when CIEDs were placed *within* the radiation field. Nevertheless, in the same phantom study, no radiation-induced effects were observed in 6 MV photon beams when a total number of 10 ICDs were placed up to 3 cm away from the PTV in clinical lung cancer SBRT scenarios [4]. In this study, specific DR effects in ICDs were not investigated.

It has been demonstrated that ICDs placed directly within the primary radiation beam malfunction [1, 5–8]. In absence of other known causes for radiation-induced CIED failures like neutron-generating photons at photon energies larger than 6 MeV, it is hypothesized that DR-related effects exist, which can influence ICD circuitry and thus lead to malfunctions. These effects may affect ICDs, which are in direct vicinity but not inside a clinical target volume. In the beam penumbra, LDR are still high but decrease rapidly with increasing distance. At present, there is no clear evidence available regarding which LDR can be safely applied and accordingly at which distance from the radiation field an ICD may be located when radiotherapy for a nearby tumor volume is of imminent need.

The aim of this study was therefore to describe ICD effects in view of specific different LDR of radiation emitted from a medical linear accelerator with and without flattening filter. ICDs were placed at predetermined distances to the primary radiation beam with known LDR within each ICD with flattened (FLAT) and FFF beams at 6 MV. This will lead to a more precise description at which LDR ICD-errors occur and which LDR may be safely applicable at the position of ICDs in patients undergoing lung SBRT. A safe distance between ICD and radiation field margin will be provided for flattened and FFF 6 MV photon beams.

## Materials and methods

### Cardiac implantable electronic devices

A total of 62 explanted ICDs (Medtronic Inc., Minneapolis and St. Jude Medical/Abbott, Saint Paul, MN, USA) were used. All devices were interrogated prior to experiments and relevant data were registered. Time between ICD implantation and radiation exposure was 4.3 ± 2.1 years. All ICDs were fully functional and had sufficient battery capacity. No ICD was previously subjected to therapeutic radiation. Detection parameters for ventricular tachyarrhythmias or pacing parameters were not reprogrammed; however, shock delivery was deactivated for safety reasons [1]. All devices were monitored continuously during experiments with wireless programmers (Medtronic, St. Jude Medical/Abbott). All abnormalities observed in real time monitoring were recorded. In addition, interrogation of each ICD took place immediately before and after each experiment.

### Experimental design and setup

**Fig. 1:**
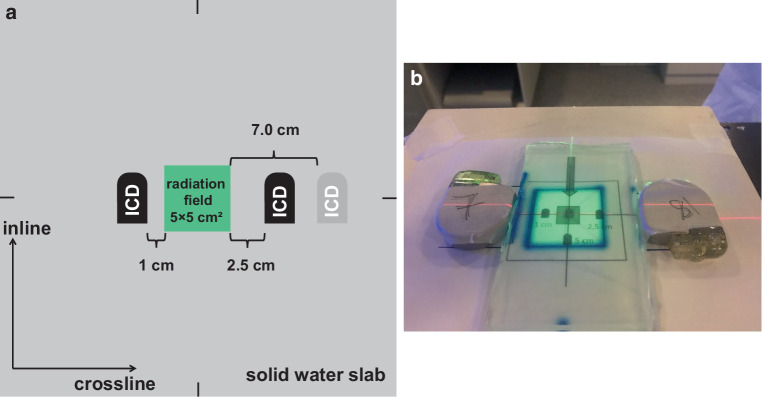
**a** Sketch of the experimental setup. Only one implantable cardioverter–defibrillator (*ICD*) was present on each side at a time; either at 1.0, 2.5, or at 7.0 cm. **b** Positioning of the ICDs with respect to the radiation field after removing the 2 cm bolus buildup-material. ICDs located at the 1 cm and 2.5 cm distance from field edge

Radiation experiments were carried out at a medical linear accelerator (LINAC; VersaHD, Elekta AB, Stockholm, Sweden). ICDs were placed on a 30 × 30 cm^2^ solid water slab phantom (RW3, PTW, Freiburg, Germany). The entire setup was covered by 2 cm bolus material (Superflab, Eckert & Ziegler, Berlin, Germany) to ensure secondary electron equilibrium. Source–surface distance (SSD) was 98 cm and the depth of the isocenter was located at the level of the ICD’s upper side. ICDs were located at predefined positions in 1.0, 2.5, and 7.0 cm distance to the nominal field edge (50% dose level) in the penumbra of the radiation field (Fig. 1) with the ICD’s inner side at the nominal distance. This setup ensured that circuitry and battery were exposed to radiation to a similar extent. For each experiment, exactly two devices were placed at opposite positions of the radiation field in crossline-direction (left–right, A–B) to ensure a comparable multileaf collimator (MLC) transmission (< 0.6%) [9] and primary beam scattered radiation. In this setup scattered radiation from opposing ICDs was considered negligible. Between the two ICDs, bolus material was placed with a size that filled the radiation field and the respective distance of each ICD to the radiation field so that all ICDs were in direct contact with the bolus material.

All dose deliveries were performed with a 5 × 5 cm^2^ radiation field. This field size was chosen since for most SBRT, equivalent square field sizes typically do not exceed a mean equivalent square of 5 cm. ICDs were subjected to scattered and MLC-transmitted radiation from 100 Gy isocenter dose. Radiation fields of 6 MV beams with a flattening filter being present and a constant dose-rate of 490 MU/min (equals 430 cGy/min) at the isocenter or a 6 MV FFF beam with a dose-rate of 1450 MU/min (equals 1400 cGy/min) at the isocenter were used.

ICDs were divided into five experimental groups with varying distances to the radiation field and irradiated with different radiation beams as described in Table 1.

**Table 1:** Beam characteristics and experimental setup of the different groups

			Group		
	1	2	3	4	5
*Beam type*	FLAT	FLAT	FFF	FFF	FFF
*Beam-on time (min)*	23.5	23.5	7.2	7.2	7.2
*Distance to radiation field (cm)*	1.0	2.5	1.0	2.5	7.0
*Number of ICDs*	10	16	10	10	16

*FLAT* flattened photon radiation, *FFF* flattening filter free photon radiation, *ICD* implantable cardioverter–defibrillator

If an ICD from groups 1, 3, and 4 showed an ICD failure, then these devices were interrogated after 8 weeks. After assertion of normal parameters, dose delivery was then repeated in these devices with the radiation beam settings of group 5 (which did not induce any ICD errors in 16 devices and exhibited the same LDR as group 2), therefore determining whether radiation-induced effects were of permanent or transient nature.

### Scattered radiation dose measurements

To determine the LDR at different distances from the radiation field, the experimental setup was reproduced with an ionization chamber at relevant positions. For the purpose of measurements, ICDs were replaced with bolus material. LDR were measured with a 0.3 cm^3^ rigid stem ionization chamber (type 30016, PTW, Freiburg, Germany) connected to an Unidos webline electrometer (PTW, Germany) after calibration to ambient temperature and pressure. Point dose measurements were performed at following positions in crossline direction: Isocenter as well as at 1.0, 2.0, 2.5, 4.0, 5.0, 7.0, 8.0, and 10 cm distance to the radiation field. The measurement setup was reproduced in the treatment planning system (TPS) Monaco (Version 5.51.10, Elekta AB). Dose calculation was Monte Carlo based with a spatial resolution of 2 mm and 1% statistical uncertainty. The reported ICD doses equate to the doses at the proximal end of the ICD and are therefore maximum doses.

### Data analysis

Any inadequate sensing (IS) ranging from a single event to continuous malfunction leading to inadequate defibrillation therapy is reported as a failure. Due to the nature of CIED events in radiotherapy and the goal to avoid any radiation-induced ICD malfunction in patients, all such ICD locations with LDR that resulted in ICD failure were deemed potentially harmful. A LDR that resulted in stable ICD function was concluded to be safe for radiotherapy. As a consequence of this dichotomic nature of our results, no subsequent data analysis (e.g., risk analysis) beyond mere data presentation was considered targeted and adequate.

## Results

### Measurement details on local dose rates

**Fig. 2:**
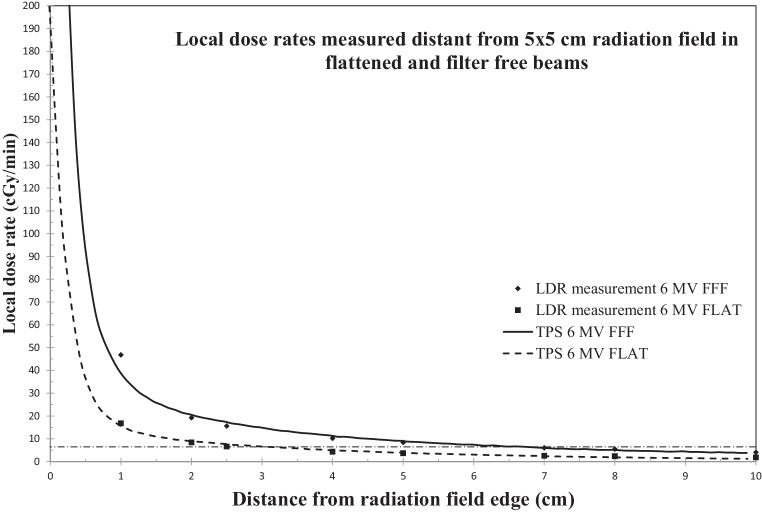
Local dose rate as a function of the distance from a 5 × 5 cm^2^ radiation field (constant dose rate in the isocenter for FLAT 430 cGy/min, FFF 1400 cGy/min). Zero distances would be at the 50% dose level at SSD = 98 cm. Measured local dose rates at specified positions and fitted curve which was received from Monte Carlo-based dose calculations. *Dashed line* denotes local dose rate of 6 cGy/min extrapolating to 2.5 cm distance from FLAT beam field edge and 7 cm distance from FFF beam field edge

Experimental groups 1 (FLAT and 1 cm distance to beam) and 4 (FFF and 2.5 cm to beam) exhibited the same LDR (16.8 vs. 15.6 cGy/min) as well as groups 2 (FLAT and 2.5 cm) and 5 (FFF and 7 cm; 6.6 vs 6.0 cGy/min; Table 2). Removal of the flattening filter (FFF) resulted at the same location (e.g., 2.5 cm distance from beam edge) in higher LDR (15.6 cGy/min) in comparison to FLAT beams (6.6 cGy/min). Of note, comparable LDR resulted in higher total ICD dose with FLAT beams in comparison to FFF (e.g., FLAT 16.8 cGy/min resulted in 3.9 Gy ICD dose, while FFF 15.6 cGy/min led to 1.1 Gy ICD dose when 100 Gy isocenter dose were delivered; Table 2). Therefore, these experiments may serve as a comparison whether the total dose or the LDR is causing malfunctions. LDR as a function of the distance from the radiation beam of 5 × 5 cm^2^ 6 MV FLAT and FFF beams at isocenter depths are shown in Fig. 2.

**Table 2:** Radiation-induced ICD failures, maximum dose, and local dose rates at the specified measurement positions

Group	1	2	3	4	5
**Maximum ICD dose (Gy)**	3.9	1.5	3.4	1.1	0.4
**Local dose rate (cGy/min)**	16.8	6.6	46.8	15.6	6.0
*Initial experiments (62 nonirradiated ICD)*					
**No failures**^a^	*n* = 8 (M, SJM)	*n* = 16 (M, SJM)	*n* = 7 (M)	*n* = 7 (M)	*n* = 16 (M, SJM)
**Failures**^a^	*n* = 2 (M, SJM)	–	*n* = 3 (M)	*n* = 3 (M)	–
*Experiments with ICD failures at higher dose rates were repeated after 8 weeks with group 5 settings*					
**No failures**^a^	*n* = 2 (M, SJM)	–	*n* = 1 (M)	*n* = 3 (M)	–
**Failures**^a^	–	–	*n* = 2 (M)	–	–

*ICD* implantable cardioverter–defibrillator

^a^ Letters in parentheses denote manufacturer: Medtronic (M); Saint Jude Medical/Abbott (SJM)

### ICD failures at described local dose rates

Results are summarized in Table 2. The following is a description of noticed ICD failures with local ICD doses and accumulated radiation doses within the isocenter at the time of malfunction. The time of first malfunction after start of the beam is provided as well. All erroneous ICDs (*n* = 8) had a time between implantation and radiation exposure of 4.1 ± 2.8 years and thus did not differ from the total collective regarding their age.

- Group 1: In the FLAT experiments, 2 of 10 ICDs which were placed at 1 cm distance from the beam malfunctioned with IS of ventricular tachycardia (VT) leading to shock (defibrillation) therapy (ISofVT-ST). The first ICD showed ISofVT-ST after 12 cGy ICD dose (isocenter dose 2.6 Gy; 31.8 s after start of the beam) with a slow recovery after 2 min. No successive errors were found until full dose was delivered. The second ICD exhibited ISofVT-ST at an ICD dose of 17 cGy (isocenter dose 4.4 Gy; after 53.9 s) for a duration of 5 s with spontaneous recovery after 5 s. This device did not exhibit any other malfunction until delivery of the full dose to the isocenter was completed. No errors were observed in these two ICDs with group 5 settings after 8 weeks.
- Group 2: No incidents were observed for 16 ICDs, which were placed 2.5 cm away from the beam.
- Group 3: In the FFF experiments, 3 of 10 ICDs which were placed at 1 cm distance from the FFF beam developed IS of atrial or ventricular signals which led to inhibition of stimulation. The first ICD showed IS after 34 cGy ICD dose (isocenter dose 10 Gy; after 41.4 s). 167 events were registered until full dose delivery. After repetition with the settings of group 5, this ICD still showed 12 events even after 8 weeks. The second ICD showed one IS at 238 cGy ICD dose (isocenter dose 70 Gy; after 289.7 s). No further events occurred when applying the group 5 settings. The third ICD showed repeatedly IS beginning with 68 cGy ICD dose (isocenter dose 20 Gy; after 82.8 s). With group 5 settings, 5 IS were observed.
- Group 4: 3 of 10 ICDs that were located at 2.5 cm distance to the FFF beam malfunctioned. The first ICD developed IS at 8 cGy ICD dose (isocenter dose 7 Gy; after 29 s). The second ICD malfunctioned with ISofVT-ST at 7 cGy ICD dose (isocenter dose 5.8 Gy; after 24 s). This particular ICD showed only ISofVT-ST when the beam was turned on and exhibited normal parameters when the beam was turned off. After full dose delivery, this ICD was interrogated and exhibited normal functional parameters. The third ICD showed IS after 22 cGy ICD dose twice (isocenter dose 20 Gy; after 82.8 s). None of the three ICDs in this group showed malfunctions when they were exposed after 8 weeks to the group 5 experimental setting after the initial group 4 dose delivery.
- Group 5: No incidents were observed for 16 ICDs, which were placed 7 cm away from the FFF beam.

## Discussion

To our knowledge, this is the first investigation which tries to identify the cause for ICD malfunctions in close vicinity of direct, non-neutron generating radiation beams when an ICD is located near but not inside the primary radiation field. ICDs exhibited transient errors when the LDR exceeded 6.6 cGy/min. Only ICDs which were exposed to a much higher LDR of 46.8 cGy/min presented persistent errors after 8 weeks. On the other hand, the total radiation dose, which an ICD sustained did not imply failures. Specifically, errors occurred when the LDR was 15.6 cGy/min and the total ICD dose was 1.1 Gy, while no errors were noted when the LDR was 6.6 cGy/min with an ICD dose of 1.5 Gy.

Previous studies have placed ICDs either inside direct radiation or at a distance where the LDR was too small to be considered a relevant factor (overview in [3]). From the presented results and considering LDR measurements at described locations, a lower limit for a LDR for ICD errors in non-neutron generating photon beams was concluded to be below 7 cGy/min for previously nonirradiated ICDs. At a LDR around 6 cGy/min, no malfunctions were observed in 32 ICDs from two different manufacturers. Malfunctions for both FFF and FLAT beams occurred at more than 15 cGy/min. In 2 of 8 ICDs, these malfunctions were persistent. In 6 of 8 ICDs, radiation-induced errors appeared to be only temporary since these devices showed no further malfunctions when they were exposed after 8 weeks to subsequent radiation with dose rates around 6 cGy/min. Furthermore, no discrimination between FLAT and FFF beams was possible with respect to quality and quantity of ICD malfunctions. However, since this comparison was conducted at points with similar mean dose rates but different distances to the radiation field no statement on varying instantaneous dose rates [10] can be drawn from these experiments. At LDR of 46.8 cGy/min repeated and persistent ICD malfunctions occurred. Even when reducing the LDR to 6 cGy/min and investigating these malfunctioning ICDs again after 8 weeks, 2 of these 3 particular ICDs showed persistent IS which indicates permanent defects in some but not all devices. For typical SBRT scenarios (reasonably small targets and thus radiation fields), it is therefore safe to keep 7.0 cm distance between ICD and the target volume for 6 MV FFF beam deliveries (even at a dose delivery with constantly 1400 cGy/min isocenter dose rate) or 2.5 cm distance of the ICD to a 6 MV FLAT beam. Larger field sizes lead to more scattered radiation, which should be reflected in the corresponding margin assignment accordingly.

The fact that ICD failures in groups 1 and 4 as well as 2 and 5 were similar in frequency and severity combined with the corresponding total ICD doses and LDR presented in Table 2 indicates that the dominant reason for ICD failures could be the LDR and not the total dose to the ICD. As visible in our subgroups, there is a more similar behavior (number of defects) in groups with similar LDR than there is in groups with similar total scatter dose. In addition, ICD errors occurred in their respective LDR groups at different cumulative radiation doses. Of note, manufacturers refrain from giving specific safe cumulative radiation doses for cardiac pacemakers and ICDs because it is currently unclear, whether there is such a cumulative dose effect or not [11]. On the other hand, all available guidelines actually express such a dose recommendation (the most used is 2 Gy) [3]. In this context we demonstrate that such a dose threshold is depending on the LDR at the position of such a device (for ICDs). We noted for a LDR of 46.8 cGy/min failures at 34, 68, and 238 cGy cumulative ICD doses. For 16.8 cGy/min, we detected failures at 12 cGy and 17 cGy cumulative ICD doses and at a comparable LDR of 15.6 cGy/min, we saw failures at 7, 8, and 22 cGy. Permanent failures, which were more severe and would have resulted in ICD replacement, were only noticed in ICDs from the 46.8 cGy/min LDR group even though these two specific ICDs failed at the lower cumulative doses of 34 and 68 cGy.

With increasing use of flattening filter free radiation techniques for SBRT for lung tumors, two factors shift in the focus with regard to radiation-induced ICD effects that were not entirely investigated in the past: high dose rates and large target volume doses. While the TG 203-report [3] states that no robust evidence exists which supports recommendations for SBRT cases in CIED patients, our recently published study [1] suggested that high dose rates may be applied in close distance to ICDs. Due to VMAT-typical variable dose rates, only few IMRT segments in the specific SBRT treatment were applied with dose-rates as high as 1500 cGy/min. Therefore, no conclusions regarding threshold-LDR were drawn from the results. Still, the data were suggestive for the notion that SBRT may be feasible even for target volumes close to an ICD because a high target volume dose of 150 Gy was applied to the isocenter with 6 MV FFF-VMAT without any ICD error.

Another recent study [4] subjected ICDs to either 6 MV or 10 MV FFF-IMRT and placed ICDs partially inside or 3 cm away from direct radiation beam. Irradiation was conducted either with 28 Gy single fraction or with 4 × 12 Gy. Here, only 10 MV plans resulted in ICD errors and no incidents were observed with 6 MV plans, therefore, corroborating that even small increments in neutrons already cause ICD upsets. In this study, an additional small number of ICDs were placed directly within radiation and remained unaffected when the dose rate was below 1200 MU/min or 1200 cGy/min for a short period.

Mouton et al. proposed a threshold dose rate of 20 cGy/min after investigating 96 cardiac pacemakers at various LDR between 0.05 and 8 Gy/min [12]. In this investigation, all pacemakers were located within the beam axis and irradiated with 18 MV photons which results in high neutron doses. Therefore, no conclusions regarding a possible safe LDR for non-neutron generating beam energies at the position of the CIED could be drawn from this particular study since neutrons remain a major cause for severe electrical upsets in CIEDs.

In view of available data, it has become clear that CIED errors occur stochastically with increasing frequencies when neutron-producing photon energies are applied [1, 13–15] or when CIEDs are placed within radiation (overview in [3]). Here, no threshold radiation ICD dose can be assumed safe because ICD errors may occur even at lowest cumulative ICD doses when placed within the beam of 6 MV beams [6].

While a local ICD dose rate of less than 1200 cGy/min may be applied for a short period [4], our data suggest in comparison that with increasing time and radiation dose, much lower dose rates already result in ICD errors. Hurkmans et al. have suggested for FFF beams, that at the location of a CIED outside of the target volume, the LDR would be lower than 100 cGy/min and therefore dose-rate effects are rare [16]. The data from Aslian et al. serve well for the explanation of several case reports of high cumulative absorbed radiation doses to CIEDs [17, 18]. The authors show that even high dose-rates of 1200 MU/min can be withstood by an ICD for a short time. On the other hand, it is possible that ICDs can withstand radiation doses that are accumulated in small increments over a longer time period [19]. Our data provide evidence for the notion that a much lower LDR of 15.6 cGy/min can result in ICD errors if sustained by the ICD for a longer period throughout one single treatment fraction, which may occur during SBRT cases. We show that ICD errors develop within the penumbra of flattened and unflattened beams and dose-rate effects are relevant. Therefore, our data substantiate the consideration that dose rate effects are rare (but exist) in clinical practice [16] because they depend on ICD position relative to the target volume.

We distributed an uneven number of ICDs among the investigated groups. After noticing ICD failures at 1 cm distance with flattened 6 MV beam (group 1), more ICDs were not included in this group because the emphasis of this investigation is focused on SBRT cases which are typically executed with FFF beams resulting in short treatment times and therefore facilitating breath-hold techniques. Furthermore, the 1 cm FFF group 3 was equipped with 10 ICDs as was the 2.5 cm FFF group 4. Here, the emphasis laid on generating a robust signal for discrimination between safe and unsafe LDR. Finally, 16 ICDs from two manufacturers were included in groups 2 and 5, corroborating our findings that LDRs of around 6 cGy/min do not result in ICD errors. Still, we investigated a total number of 62 ICDs, which is a relatively small number when looking at stochastic events. This limitation is determined by the limited availability of functioning ICDs. It might be challenging to add more measurement locations when trying to further elucidate a true threshold-LDR for ICDs (in cm steps) but this could be undertaken, after a robust power analysis using our data and when focusing on one single beam quality (flattening filter free only).

A total dose of 100 Gy at the beam isocenter is higher than any typical cumulative dose concept in lung SBRT and can therefore serve as an upper limit. An explicit consideration of potential dose fractionation effects were beyond the scope of this work, but since no ICD showed any error during maximum changes (beam-on, beam-off), a generalization to fractionated RT is considered feasible. Furthermore, any interfractional ICD recovery process will lead to fewer failures.

We included ICDs from two different manufacturers in our setting and distributed 1‑chamber, 2‑chamber and 3‑chamber devices equally among all groups, but are aware that differences in architecture of the ICDs exist between different companies. Therefore, our results cannot be generalized to all available devices. But they provide a rationale for further discussion of safe and potentially deleterious ICD locations in SBRT-planning scenarios. With the increasing use of SBRT with flattened and unflattened photon beams and high local fractional target doses, our data can help to understand deleterious dose rate effects in ICDs and provide information on how to avoid them.

## Conclusion

Dose rate effects play a role in radiation-induced ICD failures beside neutron radiation and total dose to the ICD. A LDR in ICDs between 15.6 and 6.6 cGy/min may cause ICD malfunction. At identical LDR, differences between 6 MV FFF and 6 MV FLAT beams were not found to yield different rates of malfunction. It is recommended to generate a planning risk volume (PRV) around the ICD in which LDR larger than 7 cGy/min are avoided. Depending on the effective field size and the dose rates used it is for most stereotactic treatments with 6 MV FFF beams sufficient to add a 7 cm ICD-PRV isotropic margin.
